# Curbing Inflammation in Skin Wound Healing: A Review

**DOI:** 10.1155/2015/316235

**Published:** 2015-08-18

**Authors:** Rodrigo G. Rosique, Marina J. Rosique, Jayme A. Farina Junior

**Affiliations:** Division of Plastic Surgery, Department of Surgery and Anatomy, Ribeirão Preto School of Medicine, University of São Paulo, Avenida Bandeirantes 3900, 14048-900 Ribeirão Preto, SP, Brazil

## Abstract

Wound healing is a complex regulated process that results in skin scar formation in postnatal mammals. Chronic wounds are major medical problems that can confer devastating consequences. Currently, there are no treatments to prevent scarring. In the early fetus wounds heal without scarring and the healing process is characterized by relatively less inflammation compared to adults; therefore, research aimed at reducing the inflammatory process related to wound healing might speed healing and improve the final scar appearance.

## 1. Introduction

The skin is the largest organ of the body and acts as the first line of defense against pathogens, toxins, and trauma. It also plays a critical role in fluid homeostasis and provides sensory functions and thermal regulation. Damage or loss of skin integrity resulting from an injury or disease can lead to significant morbidity and even death.

Wound healing is a complex, regulated process in which regulated collagen deposition, in response to tissue injury, results in scar formation. Its mechanisms include inflammation, fibroplasia, and scar maturation.

Sometimes cutaneous wounds do not progress to normal healing with formation of a final mature scar formation but to a continuing inflammatory process, which can lead to a more aggressive carcinogenic transformation in long time of evolution (Marjolin's Ulcer). Many chronic wounds are the result of chronic inflammation. In contrast to adult wound healing, the early gestation fetus displays a remarkable ability to heal wounds without scarring. Fetal wounds heal rapidly and are characterized by a relative lack of inflammation [1]. The introduction of inflammation into normally scarless wounds produces scarring [2]. Conversely, reduction of inflammation in postnatal wounds can reduce scarring [3].

In this paper, we review how to curb inflammation in cutaneous wound healing; the following lists the main topics discussed in this paper.

*Topics about Curbing Inflammation in Wound Healing*

*General Topics*

- Wet environment.
- Primary closure and sutures.
- Drugs or supplement:
  - curcumin,
  - protein supplementation,
  - nutraceuticals,
  - neurotensin (NT),
  - photodynamic therapy (PDT),
  - doxycycline,
  - tumour necrosis factor-alpha (TNF-*α*) antibody.
- Technology:
  - transcutaneous electrical nerve stimulation (TENS),
  - hyperbaric oxygen,
  - silver dressings,
  - negative pressure wound therapy (NPWT).
- Biomaterials and cellular therapy.

*Specific Wounds*

- Diabetic wounds.
- Vascular wounds.
- Irradiated wounds.

## 2. Postnatal Wound Healing Process

Repair of the skin consists of three phases: an initial inflammatory phase and then a proliferative/repair phase and it concludes with a remodeling phase, which results in scar formation in postnatal mammals.

In response to tissue injury, inflammatory cells are recruited to wounded tissue. The acute inflammatory response is followed by proliferation of fibroblasts, which are responsible for synthesizing collagen and extracellular matrix. Fibroblasts can differentiate into myofibroblasts, which are responsible for collagen deposition and wound contraction. Ultimately, a scar results from accumulation of extracellular matrix. Despite scar remodeling during maturation, normal architecture is never completely restored [4]; only 70 percent of the tensile strength of normal skin is recovered [5].

The early stage of inflammation is regarded as a critical period of the wound healing process, essential for clearing contaminating bacteria and creating an environment conducive to the succeeding events of tissue repair and regeneration [6]. The injury causes a gap, which is immediately filled by clots in the presence of platelet aggregates. Then, during the inflammatory phase, leukocytes, such as neutrophils, monocytes, and macrophages, infiltrate the site, remove the breakdown products of the injured cells and clots, and release various growth factors and cytokines [7]. In response to growth factors and cytokines, the proliferative phase starts. In this phase, epidermal cells migrate, proliferate to fill the wound gap, and displace remnants of the original clots. Thus, it is generally accepted that leukocyte and macrophage infiltration is essential to wound healing.

Cytokines have been widely studied because they are important to wound healing; they regulate the activity of the cells that produce the healing response to tissue injury [8]. Several cytokines, including interleukin- (IL-) 1*β* and tumor necrosis factor- (TNF-) *α*, have been shown to regulate the recruitment and function of neutrophils. In irradiated mice, an investigation of the ability of exogenous IL-1*β* or transforming growth factor-*β* to reverse radiation-induced defective wound healing found that IL-1*β* enhanced wound tensile strength [9]. TNF-*α* is a major cytokine secreted by macrophages and neutrophils during the inflammation phase of wound healing; it is elevated in early wound healing [10].

In all phases of wound repair, extracellular matrix (ECM) proteins play a key role in directing the fate and activities of progenitor and reparative cells. Immediately after injury, the ECM orchestrates the recruitment of platelets and directs the inflammatory cell response that initiates the hemostatic and the cellular debridement phases [11]. These cells, which migrate into the wound bed of the ECM of the initial hemostatic plug and then migrate into the provisional matrix, respond to individual ECM components and growth factors (which may be bound to this matrix). These cells, in turn, recruit and direct stem/progenitor and reparative cells from both distant and local sites to mediate the proliferative/repair phase of healing. Particularly, in this rebuilding phase of healing, adult stem cells participate critically in replenishing cells that were damaged or lost after injury. In addition to their role after trauma, adult stem cells participate in the maintenance of the skin as well as wound healing [12].

## 3. General Topics

### 3.1. Wet Environment

A wet or moist environment in wounds has been shown to promote reepithelialization and result in reduced scar formation more than a dry environment [13]. The inflammatory reaction is reduced in the wet environment, thereby limiting injury progression. Several studies have compared wet, moist, and dry healing. A wet or moist incubator-like microenvironment achieves fastest healing with fewest aberrations and least scar formation.

The modern approach of employing a moist environment for the treatment of wounds was introduced in the early 1960s by Winter [14], who showed in a pig model that the rate of epithelialization after wounding was doubled by using a moist dressing as compared to dry conditions. This was a new concept that opposed the generally accepted idea that a dry environment could best fight wound infection.

Manufacturers responded to Winter's research findings and provided a wide range of moist dressings, such as hydrocolloids that absorb the wound fluid beneath a semiocclusive dressing [15], foams [16, 17], alginates [18], and hydrogels [19, 20]. Using the Cochrane database, Dumville et al. [21–24] performed systematic reviews of the four types of wound dressings to evaluate their contribution to the healing of diabetic ulcers. A systematic review by Wiechula [25] suggests that moist wound healing products have distinct clinical advantages over dry products for the management of split-thickness skin donor sites.

Adult human skin wounds heal with varying degrees of scar formation [26]. Scarring is correlated with the intensity and duration of inflammation during healing [27]. In 2009, Reish et al. [28] published an experimental study of porcine wounds treated either in a moist environment or with gauze: inflammation was compared by evaluating the number of inflammatory cells/high-power field 3 days after wounding. Inflammation was greatly reduced in the wounds treated in the wound chamber, and the number of inflammatory cells on day 3 was very strongly correlated with the amount of scarring at day 28. Compared with dry wounds, wet wounds created under sterile conditions and treated with low concentrations of antibiotics exhibited a significantly smaller macroscopic scar surface area in all experimental wound groups. Wounds with a greater inflammatory cell infiltrate could be predicted to develop more residual scarring.

### 3.2. Primary Closure and Sutures

Surgical sutures are used universally to achieve proper wound approximation by supporting the strain of closure until sufficient healing has occurred. Approximating the wound wedges with sutures decreases the wound area subjected to the healing process and abbreviates the inflammatory phase, which decreases scarring due to the faster pace of healing. All sutures are foreign bodies; therefore their presence triggers a local immune response. Thereby sutures may, paradoxically, potentiate and extend the inflammatory phase of wound healing, resulting in undesirable outcomes, such as wound breakdown, suture “spitting,” hypertrophic scar, or keloid formation. The degree of inflammation induced varies with suture composition, which ranges from relatively inert polypropylene and nylon to highly inflammatory silk and plain gut suture. As a result, numerous investigations have attempted to alter suture composition with the goal of reducing the inflammatory response; however, no specific composition has gained clinical relevance or widespread practice [29].

### 3.3. Drugs or Supplement

#### 3.3.1. Curcumin

Kant et al. [30] found that curcumin, an anti-inflammatory and antioxidant agent, caused faster and better wound healing in diabetic rats. This effect was attributed to a decreased expression of inflammatory cytokines and enzymes, TNF-*α*, IL-1*β*, and MMP-9, and increased levels of the anti-inflammatory cytokine IL-10 and antioxidant enzymes, SOD, catalase, and GPx, at the wound site in diabetic rats. This data allows us to suggest that curcumin could be an additional novel therapeutic agent for the management of impaired wound healing in diabetics and radiated tissues [31, 32].

#### 3.3.2. Protein Supplementation

Abdel-Salam [33] demonstrated that whey (milk serum) protein could contribute to cutaneous wound healing. In this investigation, wound closure was significantly delayed in the diabetic group. Moreover, the results clearly demonstrated that whey protein supplementation of diabetic animals enhanced wound closure and restored proinflammatory (IL-1*β*, IL-6, and TNF-*α*) and anti-inflammatory cytokine (IL-4 and IL-10) levels nearly to the levels of control animals. These findings support the hypothesis that delayed wound healing in diabetic animals is caused by deregulation of signaling molecules that are critical mediators of the inflammatory response.

#### 3.3.3. Nutraceuticals

Serra et al. [34] evaluated the effects of a new nutraceutical on both clinical and molecular parameters in patients with chronic vascular ulcers, since bioflavonoids were shown to have provessel activities along with anti-inflammatory, antioxidant, and phlebotonic effects in the treatment of chronic venous disease. They found that it decreases inflammatory cytokine, MMPs and NGAL, levels and both improved symptoms and accelerated wound healing.

#### 3.3.4. Neurotensin (NT)

Based on findings that peripheral nerves and cutaneous neurobiology contribute to correct wound healing [35], Moura et al. [36] studied the effect of neurotensin (NT), a neuropeptide that acts as an inflammatory modulator in wound healing. They prepared a collagen-based dressing as vehicle to deliver NT, applied it to diabetic mice, and found that it can effectively support NT's release into diabetic wounds, enhancing the healing process. Nevertheless, compared to treatment with NT alone, a more prominent scar was observed in diabetic wounds treated with the collagen-based dressing.

#### 3.3.5. Photodynamic Therapy (PDT)

Reports of animal studies indicate that photodynamic therapy (PDT) improves healing of excisional wounds but the mechanism is uncertain. Mills et al. [37] first reported the effect of PDT on healing of acute excisional wounds in humans. They showed that treatment with methyl aminolevulinate- (MAL-) PDT modulated clinical and microscopic indicators of healing and, ultimately, produced scars with improved dermal matrix architecture. Pivotally, the number of TGF-b3-producing cells was significantly higher in MAL-PDT-treated wounds than in controls after 3 weeks, with an elevated TGF-b3 : b1 ratio. At 9 months, MAL-PDT-treated wounds showed improved deposition and organization of dermal matrix protein at the histological level. Thus, this regimen of PDT of wounds appears to mediate an antiscarring phenotype, with TGF-b3 as a potential key modulator.

#### 3.3.6. Doxycycline

Serra et al. [38] documented that doxycycline improved healing of chronic venous ulcers. In their study, oral low doses of doxycycline 20 mg were administrated for 3 months to a group of patients. The treated patients showed a higher healing rate compared to control group. Doxycycline has anti-inflammatory and immunomodulatory effects, through the inhibition of metalloproteinases, representing a potential solution to support wound healing.

#### 3.3.7. Tumour Necrosis Factor-Alpha (TNF-*α*) Antibody Infliximab

Therapeutic antibodies such as infliximab can inhibit TNF-*α* activity. In a case series [39], infliximab was applied topically to eight patients with 14 chronic ulcers of more than 4-month duration, which failed to respond to any previous conventional treatment. Infliximab was applied repeatedly to ulcers either as a 10 mg/mL solution or as a gel formulation (0.45, 1, or 4.5 mg/g). The authors observed, after 8 weeks, that five ulcers completely healed, while another four were reduced by more than 75% in size.

### 3.4. Technology

#### 3.4.1. Transcutaneous Electrical Nerve Stimulation (TENS)

Transcutaneous electrical nerve stimulation (TENS) has been shown to accelerate the healing of chronic wounds in human subjects and induced wounds in animal models [40, 41]. More specifically, it has been shown to significantly increase the rate of wound epithelialization [40] and contraction [42]. Kutlu et al. [43] demonstrated that TENS of incised wounds improves healing by increasing growth factors (epidermal growth factor, platelet-derived growth factor-A, and fibroblast growth factor-2) in the dermis and epidermis. In a later study, Gürgen et al. [44] showed marked decreases in the levels of proinflammatory cytokines in the dermis of the TENS-treated group, which suggests that TENS shortened the healing process by inhibiting the inflammation phase.

#### 3.4.2. Hyperbaric Oxygen

Hyperbaric oxygen therapy (HBOT), an effective tool that helps skin wound healing, is defined as delivery of 100% oxygen at greater than one atmospheric pressure (ATA) into the core of the wound [45]. Delivery of this increased amount of oxygen to the cells of unhealed tissues expedites healing of subacute and chronic cutaneous wounds [46].

Hyperbaric oxygen works by the following four dominant mechanisms:

(1) Augmented hydroxylation: it helps improve collagen synthesis.
(2) Angiogenesis: HBOT creates a large oxygen gradient between the center and periphery of the wound that strongly stimulates angiogenesis, which is seminal for wound healing [47].
(3) Increased bactericidal activity.
(4) Increased collagen deposition.

Kalani et al. showed that when diabetic foot ulcers were treated with HBOT, after 3 years of therapy, they healed completely in 76% of patients, whereas the ulcers of only 48% patients healed completely with conventional treatment [48]. In a randomized study by Kessler et al., either HBOT or standard treatment was given to 28 hospitalized patients with neuropathic ulcers (Wagner grades I to III). After two weeks of treatment, the ulcer area was reduced by half in the HBOT group (41.8 ± 25.5 versus 21.7 ± 16.9%, *P* = 0.037). This improvement, however, disappeared at the two-week follow-up [49].

#### 3.4.3. Silver Dressings

Ionized silver (Ag+) has both anti-inflammatory and antimicrobial properties with a broad spectrum of action [50–52]. Jemec et al. [53] found that treatment with silver dressings during the initial four weeks produced a total cost saving compared with treatment with nonsilver dressings. In addition, the wounds of patients treated with silver dressings closed faster than those treated with nonsilver dressings. Bisson et al. [54] found that silver dressings produced significant anti-inflammatory effects in chronic skin inflammation. It is believed that silver can improve wound healing due to its antimicrobial and anti-inflammatory properties.

Wu et al. [55] found that nanocrystalline silver can also reduce inflammation and promote scald wound healing in animal models. Sibbald et al. [56] concluded that evidence indicates that nanocrystalline silver dressings may reduce bacterial levels, decrease the chronic inflammatory response, and thus promote wound healing. Likewise, healing of chronic venous leg ulcers was associated with less wound bacteria and less neutrophilic inflammation but was associated with persistent or high lymphocyte count; greater numbers of lymphocytes were associated with more reduction in ulcer size.

#### 3.4.4. Negative Pressure Wound Therapy (NPWT)

According to prospective and retrospective clinical and experimental studies [57, 58], negative pressure wound therapy (NPWT) has been widely used to facilitate healing of acute and chronic wounds. This therapy has been shown to provide a moist wound healing environment, increase granulation tissue formation, reduce edema, and stimulate angiogenesis and blood flow to the wound margins [59–63]. In a retrospective clinical study, Torbrand et al. [64] attributed these biological effects to the evenly distributed transduction of negative pressure to the wound bed by a vacuum pump.

The impact of NPWT on local expression of proinflammatory cytokines, the number of neutrophils, and the bacterial bioburden living on the wound surface suggest that NPWT of acutely infected soft-tissue wounds leads to increased local IL-1*β* and IL-8 expression in the early phase of inflammation, which may trigger infiltration of neutrophils and thus accelerate bacterial clearance. Furthermore, the success of NPWT of acute wounds can attenuate the expression of TNF-*α*, and this effect may partly explain why NPWT does not significantly impair wound healing [65].

NPWT not only enhances the granulation tissue in the more superficial layer of the wound bed but also appears to affect deeper layers, with relief of chronic inflammation and tissue stabilization. It is believed that NPWT removes excess fluid and thereby removes proteolytic enzymes that negatively influence the healing process [66].

Interestingly, studies have shown that the activity of some matrix metalloproteinases (MMPs) is elevated in chronic wounds and the presence of these molecules is related to impairing wound healing. For instance, high concentrations of MMP-9 and high MMP-9 : tissue inhibitors of MMP (TIMP-1) ratio in wound fluid predict poor wound healing in diabetic foot ulcers [67]. In addition, in chronic wound exudates, mainly MMP-1 type collagenase is present [68]. These findings may partially explain the cause of difficult healing in chronic wounds.

On the other hand, data strongly suggest that NPWT influences the microenvironment of the wound by reducing the levels of MMPs and the MMP : TIMP ratio. Mouës et al. [69] found significantly lower levels of pro-MMP-9 and a lower total MMP-9 : TIMP-1 ratio in TNP-treated wounds during 10 days of follow-up. Stechmiller et al. showed that NPWT-treated wounds had decreased levels of MMP-3 and MMP-9 and lower MMP-3 : TIMP-1 ratios in wound fluid from pressure ulcers [70]. NPWT may lower collagenase activity and thereby prevent exaggerated degradation of collagen and promote wound healing.

**Figure 1:**
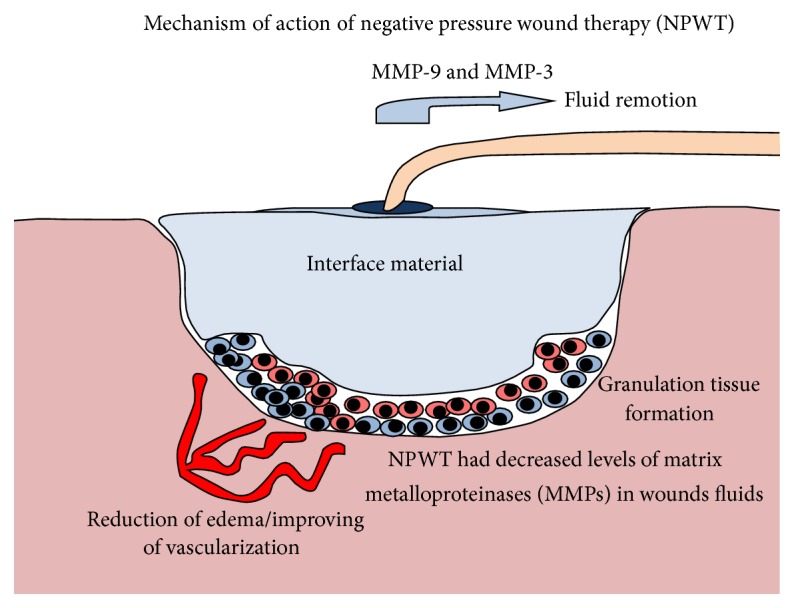
Demonstration of the principal mechanisms of action attributed to NPWT.

In addition, some studies compared different types of interface material for the application of NPWT with other common dressings. Tuncel et al. [71], who studied dressings applied to rabbit wounds, found NPWT superior to control saline-moistened gauze dressing but found no difference among three different interface materials (polyurethane foam, saline soaked antimicrobial gauze, and loofah sponge) used in association with NPWT (Figure 1).

### 3.5. Biomaterials and Cellular Therapy

The use of acellular and cell-based tissue-engineered dermal substitutes has become increasingly routine [72]. Dermal matrices and other biomimetic scaffolds for delivery of adult stem cells have been shown to augment the regenerative potential of mesenchymal stem cells (MSCs) and enhance wound healing through their presumptive ability to recreate a regenerative niche [73–75]. Besides their ability to improve the efficiency and quality of cutaneous wound healing, existing products have not yet been able to promote repair that approximates uninjured skin. The continuous evolution of superior biomaterials will depend upon a greater understanding of the role that individual components play in the regenerative niches of uninjured and injured skin, particularly the ECM constituents. Further, the development of successful biomimetic and bioresponsive substrates should incorporate the knowledge of not only how cells interact with these substrates but also how these cells then respond by remodelling and depositing their own ECM.

**Figure 2:**
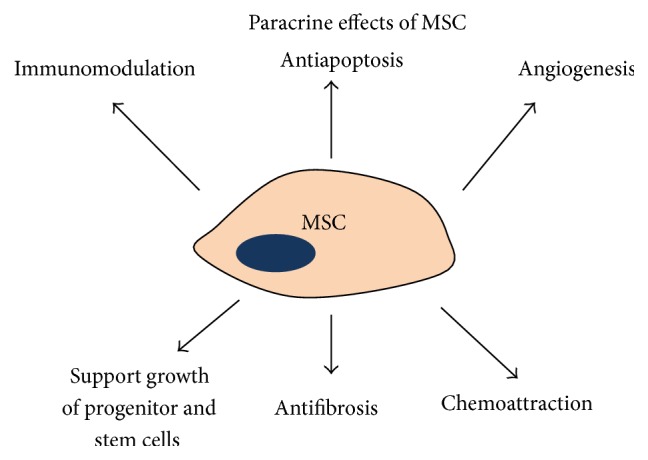
Paracrine effects of cultured mesenchymal stem cells (MSCs). It is now believed that the secretion of a wide variety of bioactive molecules may be the major mechanism by which CMMs achieve their therapeutic effect. This mechanism can be categorized into these six major actions: immunomodulatory, antiapoptosis, angiogenesis, support of the growth and differentiation of stem cells and progenitor sites, scarring modulation, and chemotaxis [78].

Of note, tissue engineering and regenerative medicine have shown significant promise in treatment of problematic wounds. Initial proof-of-principle studies that investigated the use of exogenously applied adult stem cells for the treatment of chronic wounds provided support for their vulnerary effects [76, 77]. MSCs have been shown to improve wound healing through their ability to directly contribute cells and ECM components to the repair process, as well as their ability to direct other cells participating in the repair process through their production of paracrine mediators (Figure 2) [78]. Human MSCs appear to have potent immune regulatory actions that make them attractive for use in human diseases that involve tissue injury and/or inflammation. Although initial attempts with injection of cells alone showed improved healing, it is clear that delivery strategies that reproduce complex stem cell niches will be required to maximize their potential. Given the ability of the ECM to influence many key aspects of that niche, such as signaling events, regulation of growth factor bioavailability, and mechanosensation, the identification of specific ECM components that most accurately recreate a functional stem cell niche for incorporation into therapeutic biomaterials remains a focus of intense investigation.

## 4. Specific Wounds

### 4.1. Diabetic Wound

Diabetes is a multisystem disorder, and its complications induce physiological changes in tissues and cells that impair the normal healing process. However, the pathophysiologic relationship between diabetes and impaired healing is complex. Diabetic wounds become halted in the inflammatory phase, which is characterized by a continuing influx of neutrophils that release cytotoxic enzymes, free radicals, and inflammatory mediators that cause extensive collateral damage to surrounding tissue. These destructive processes counteract the healing process in such wounds, and the overproduction of free radicals, which induce oxidative stress, results in detrimental cytotoxic effects that delay wound healing [79, 80].

Increased oxidative stress is one of the most common reasons for the delayed wound healing in the diabetic population [81]. Therefore, reduction/termination of the persistent inflammation and elimination of free radicals by the introduction of anti-inflammatory agents and antioxidants into the treatment of wounds could be an important strategy to improve healing of diabetic wounds [80].

### 4.2. Vascular Wound

Chronic venous, lower limb ulceration (VLU) affects 1–3% of the adult population worldwide, [82] and some patients suffer repeated cycles of ulceration, healing, and recurrence. The underlying pathogenesis of these hard-to-heal VLUs is complicated by excessive and prolonged inflammation that is often related to critical microbial colonization and early localized infection [83, 84]. A heavy bioburden of colonizing microorganisms in the wound may be one of the most important barriers to wound closure [85].

Pathophysiological events involved in the onset of chronic venous ulceration are inflammation, activation of polymorphonucleates (PMNs), and secretion of proteases such as MMPs, which degrade the ECM that supports the vascular and tissue walls. MMPs, neutrophil gelatinase-associated lipocalin (NGAL), and inflammatory cytokines are overexpressed in VLUs, and they could play a central role in pathophysiological mechanisms of skin lesions and delayed wound healing.

### 4.3. Irradiated Wound

Approximately 50% of cancer patients receive radiation as an adjuvant therapy. Although radiation therapy technology has progressed substantially in the last decades, patients still suffer from various degrees of nonspecific radiation damage to noncancerous tissues. Radiation ulcers have posed an enormous challenge to reconstructive surgeons; also, they cause great distress to patients, impair their quality of life, and consume high amounts of medical resources. These kinds of chronic wounds can last for several years, and some may even lead to amputations. Strategies for treating these and other chronic wounds may include the use of various growth factors [86–88], hyperbaric oxygen [89], and stem cells [90].

## 5. Conclusion

Over the last fifty years, the complex wound healing process became clearer allowing the development of strategies, devices, and medications that help achieve a better final scar, mainly through strategies to curb the wound-related inflammation. Nonetheless, we are still far from definitely solving the chronic wounds or the pathologic scarring issue and, even more, achieving the goal of rapid and scarless healing similar to fetal one.
